# Supplementary figures and images for: Doublecortin and Glypican-2 concentrations in the cerebrospinal fluid from infants are developmentally downregulated
Source: PLoS One. 2023 Feb 17;18(2):e0279343. doi: 10.1371/journal.pone.0279343 (PMC9937498; doi:10.1371/journal.pone.0279343)

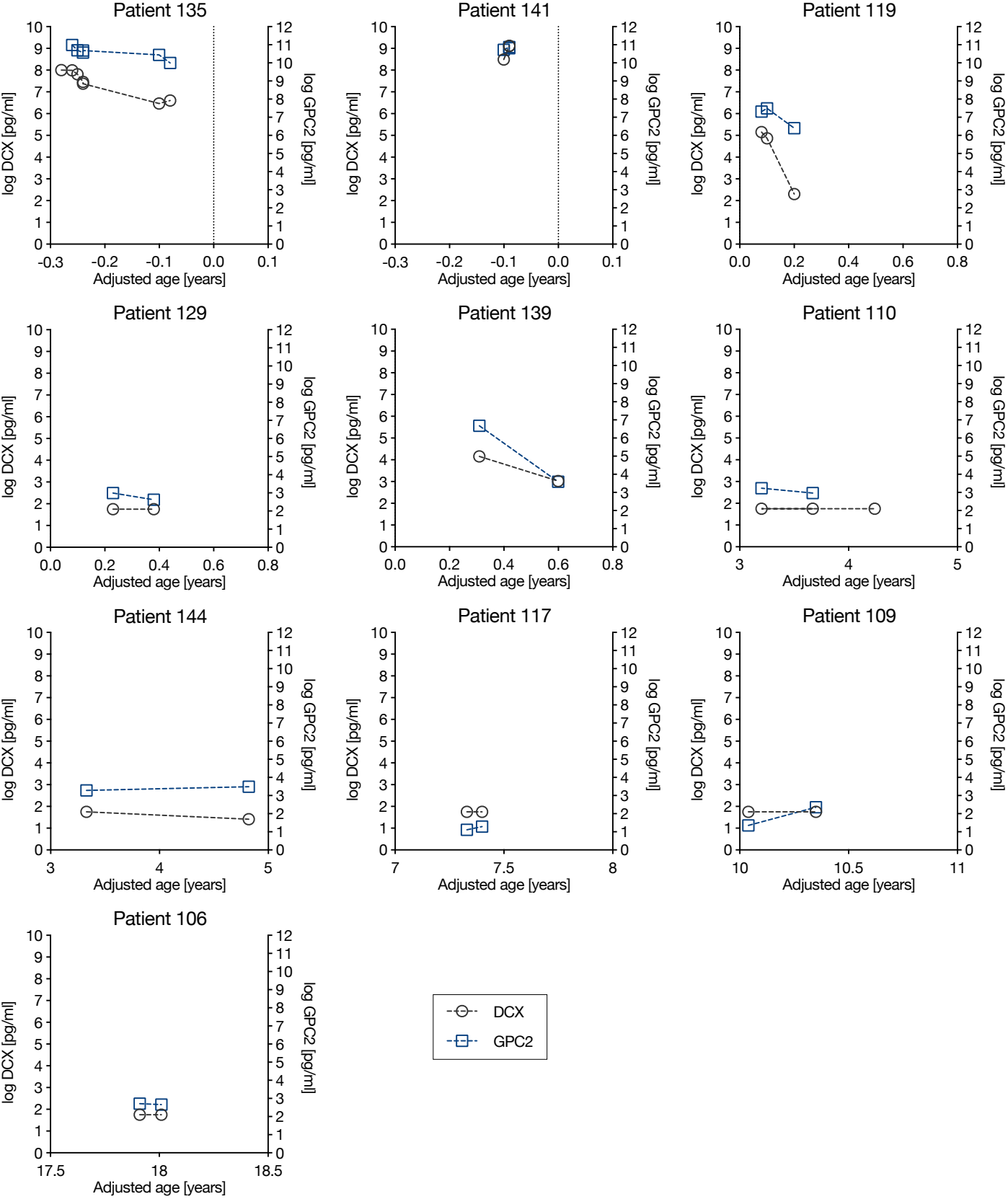

Supplement: S1 Fig — (PDF) [file pone.0279343.s001.pdf]

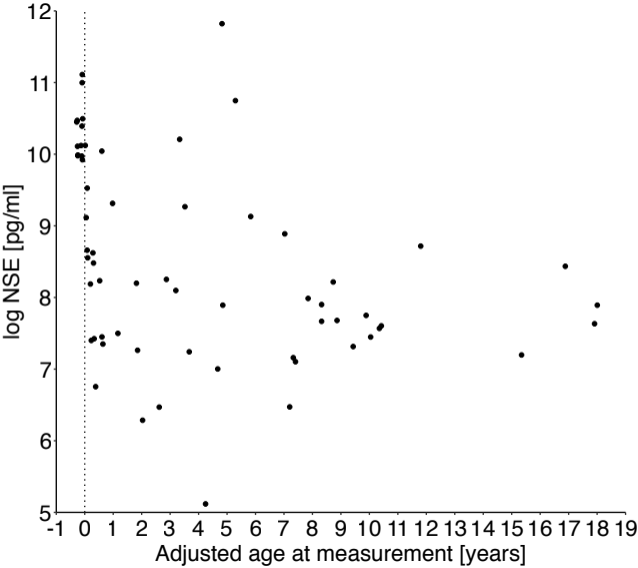

Supplement: S2 Fig — (PDF) [file pone.0279343.s002.pdf]

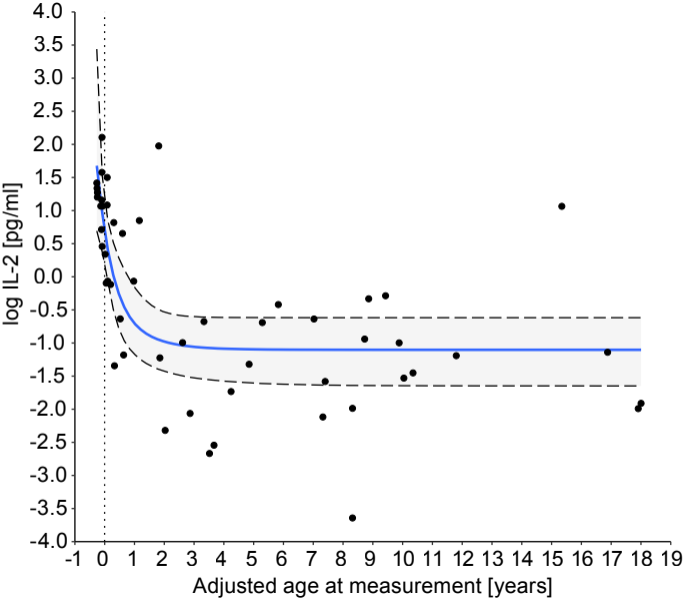

Supplement: S3 Fig — (PDF) [file pone.0279343.s003.pdf]

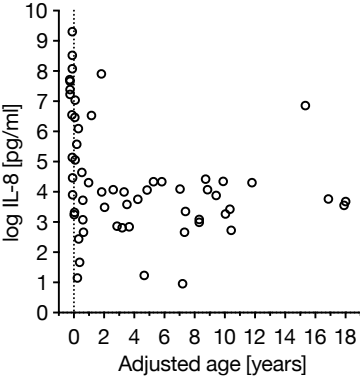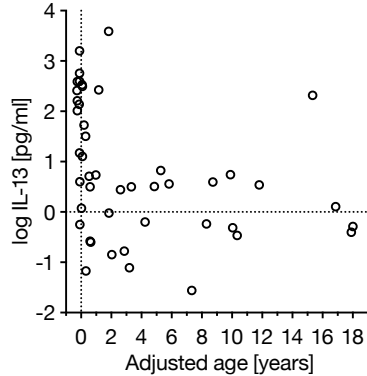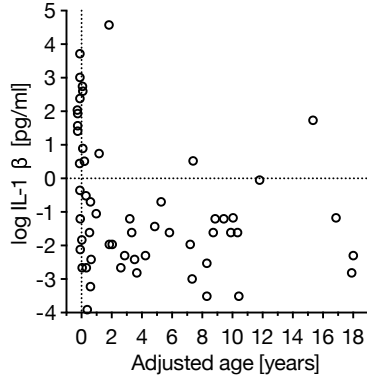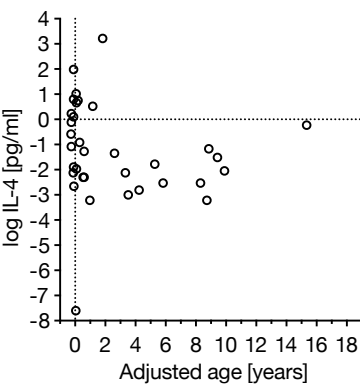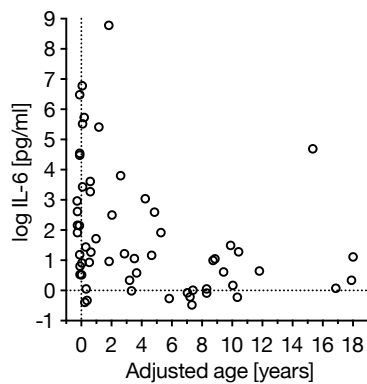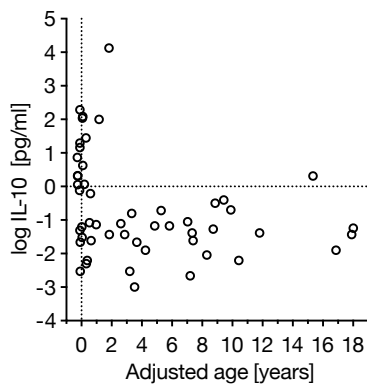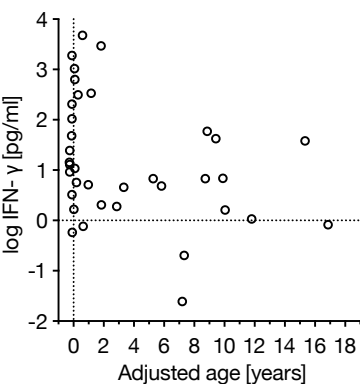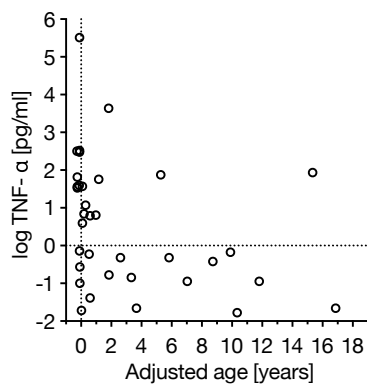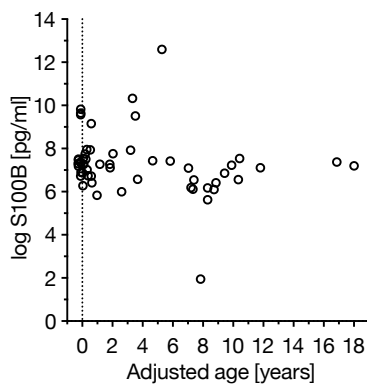

Supplement: S4 Fig — (PDF) [file pone.0279343.s004.pdf]
