# Supplementary material for: Doublecortin and Glypican-2 concentrations in the cerebrospinal fluid from infants are developmentally downregulated
Source: PLoS One. 2023 Feb 17;18(2):e0279343. doi: 10.1371/journal.pone.0279343 (PMC9937498; doi:10.1371/journal.pone.0279343)
Supplement: S1 Table — (PDF) [file pone.0279343.s005.pdf]

S1 Table. Detailed clinical baseline characteristics of all 38 patients for each CSF sample.

Sample	Patient number	Sex	Preterm birth	Adjusted age (years)	Origin of CSF sample	Indication for CSF puncture	Main diagnosis	Main Diagnosis categorized	Infection at time of CSF collection	Hydro cepha-lus
**1**	102	Female	No	15.35	Ventricular	CSF drainage	Ruptured arteriovenous malformation	Other	No	Yes
**2**	103	Male	No	1.17	Ventricular	CSF drainage	Posterior fossa ependymoma WHO II	Tumor	Yes	Yes
**3**	104	Male	Yes	7.03	Spinal	Intrathecal baclofen pump	X-linked hydrocephalus syndrome	Other	No	Yes
**4**	106	Male	No	17.91	Spinal	Intrathecal baclofen pump	Quadriplegic cerebral palsy with hypoxic encephalopathy	Spastic cerebral palsy	No	No
**5**	106	Male	No	18.01	Spinal	Intrathecal baclofen pump	Quadriplegic cerebral palsy with hypoxic encephalopathy	Spastic cerebral palsy	No	No
**6**	107	Male	Yes	-0.07	Ventricular	CSF drainage	Congenital aqueductal stenosis	Congenital aqueductal stenosis	No	Yes
**7**	107	Male	Yes	2.03	Ventricular	CSF drainage	Congenital aqueductal stenosis	Congenital aqueductal stenosis	No	Yes
**8**	108	Male	No	0.29	Arachnoid cyst	Arachnoid cyst drainage	Symptomatic arachnoid cyst	Symptomatic arachnoid cyst	No	No
**9**	108	Male	No	0.98	Arachnoid cyst	Arachnoid cyst drainage	Symptomatic arachnoid cyst	Symptomatic arachnoid cyst	No	No
**10**	109	Female	Yes	10.04	Spinal	Intrathecal baclofen pump	Spastic cerebral palsy	Spastic cerebral palsy	No	No

**11**	109	Female	Yes	10.35	Spinal	intrathecal baclofen pump	Spastic cerebral palsy	Spastic cerebral palsy	No	No
**12**	110	Female	No	3.67	Ventricular	CSF pressure measurement	Hydrocephalus malresorptivus of unknown cause	Hydrocephalus malresorptivus	No	Yes
**13**	110	Female	No	3.20	Ventricular	CSF drainage	Hydrocephalus malresorptivus of unknown cause	Hydrocephalus malresorptivus	No	Yes
**14**	110	Female	No	4.24	Ventricular	CSF drainage	Hydrocephalus malresorptivus of unknown cause	Hydrocephalus malresorptivus	No	Yes
**15**	111	Female	No	11.80	Arachnoid cyst	Arachnoid cyst drainage	Symptomatic arachnoid cyst	Symptomatic arachnoid cyst	No	Yes
**16**	113	Male	Yes	2.87	Ventricular	CSF drainage	Congenital aqueductal stenosis	Congenital aqueductal stenosis	No	Yes
**17**	114	Female	No	4.85	Spinal	Biopsy	Langerhans Cell histiocytosis	Other	No	No
**18**	116	Female	No	7.20	Ventricular	CSF drainage	Tectal glioma with hydrocephalus	Tumor	No	Yes
**19**	117	Female	No	7.33	Spinal	Intrathecal baclofen pump	Tetraspastic cerebral palsy with perinatal asphyxic encephalopathy	Spastic cerebral palsy	No	No
**20**	117	Female	No	7.40	Spinal	Intrathecal baclofen pump	Tetraspastic cerebral palsy with perinatal asphyxic encephalopathy	Spastic cerebral palsy	No	No
**21**	118	Male	Yes	16.88	Spinal	Intrathecal baclofen pump	Spastic cerebral palsy with hypoxic encephalopathy	Spastic cerebral palsy	No	No
**22**	119	Male	Yes	0.08	Ventricular	CSF drainage	Hydrocephalus malresorptivus, post intraventricular hemorrhage	Hydrocephalus malresorptivus	Yes	Yes
**23**	119	Male	Yes	0.10	Ventricular	CSF drainage	Hydrocephalus malresorptivus, post intraventricular hemorrhage	Hydrocephalus malresorptivus	No	Yes

**24**	119	Male	Yes	0.20	Ventricular	CSF drainage	Hydrocephalus malresorptivus, post intraventricular hemorrhage	Hydrocephalus malresorptivus	No	Yes
**25**	120	Female	No	1.86	Ventricular	CSF drainage	Blake pouch cyst with hydrocephalus	Other	No	Yes
**26**	121	Female	No	0.02	Ventricular	CSF drainage	Congenital aqueductal stenosis	Congenital aqueductal stenosis	No	Yes
**27**	121	Female	No	0.64	Ventricular	CSF drainage	Congenital aqueductal stenosis	Congenital aqueductal stenosis	No	Yes
**28**	123	Male	No	0.33	Ventricular	Arachnoid cyst drainage	Symptomatic arachnoid cyst	Symptomatic arachnoid cyst	No	No
**29**	124	Male	No	1.82	Spinal	Wound revision	Wound infection after spinal lipoma surgery	Other	Yes	No
**30**	125	Female	Yes	-0.13	Ventricular	CSF drainage	Hydrocephalus malresorptivus, post intraventricular hemorrhage	Hydrocephalus malresorptivus	No	Yes
**31**	125	Female	Yes	0.09	Ventricular	CSF drainage	Hydrocephalus malresorptivus, post intraventricular hemorrhage	Hydrocephalus malresorptivus	No	Yes
**32**	126	Female	No	0.05	Ventricular	CSF drainage	Myelomeningocele with hydrocephalus	Other	No	Yes
**33**	127	Female	No	2.62	subdural	CSF drainage	Bilateral hygroma post epilepsy surgical hemispherotomy	Other	No	No
**34**	128	Female	No	8.32	Spinal	Intrathecal baclofen pump	Suspected hereditary spastic paraparesis	Other	No	No
**35**	128	Female	No	8.32	Spinal	Intrathecal baclofen pump	Suspected hereditary spastic paraparesis	Other	No	No
**36**	129	Male	No	0.23	Ventricular	CSF drainage	Crouzon syndrome	Other	No	Yes
**37**	129	Male	No	0.38	Ventricular	CSF drainage	Crouzon syndrome	Other	No	Yes

**38**	130	Male	No	3.52	Ventricular	Biopsy	Tectal glioma with hydrocephalus	Tumor	No	Yes
**39**	131	Female	Yes	0.61	Ventricular	CSF drainage	Crouzon syndrome	Other	No	Yes
**40**	132	Female	No	0.53	Ventricular	CSF drainage	Congenital communicating hydrocephalus	Other	No	Yes
**41**	133	Male	No	7.85	Spinal	Intrathecal baclofen pump	Spastic cerebral palsy with perinatal hypoxic encephalopathy	Spastic cerebral palsy	No	No
**42**	133	Male	No	8.73	Spinal	Intrathecal baclofen pump	Spastic cerebral palsy with perinatal hypoxic encephalopathy	Spastic cerebral palsy	No	No
**43**	134	Female	Yes	5.83	Spinal	Intrathecal baclofen pump	Spastic cerebral palsy with perinatal intraventricular hemorrhage and hydrocephalus	Spastic cerebral palsy	No	Yes
**44**	135	Female	Yes	-0.28	Ventricular	CSF drainage	Hydrocephalus malresorptivus, post intraventricular hemorrhage	Hydrocephalus malresorptivus	No	Yes
**45**	135	Female	Yes	-0.26	Ventricular	CSF drainage	Hydrocephalus malresorptivus, post intraventricular hemorrhage	Hydrocephalus malresorptivus	No	Yes
**46**	135	Female	Yes	-0.25	Ventricular	CSF drainage	Hydrocephalus malresorptivus, post intraventricular hemorrhage	Hydrocephalus malresorptivus	No	Yes
**47**	135	Female	Yes	-0.24	Ventricular	CSF drainage	Hydrocephalus malresorptivus, post intraventricular hemorrhage	Hydrocephalus malresorptivus	No	Yes
**48**	135	Female	Yes	-0.24	Ventricular	CSF drainage	Hydrocephalus malresorptivus, post intraventricular hemorrhage	Hydrocephalus malresorptivus	No	Yes
**49**	135	Female	Yes	-0.10	Ventricular	CSF drainage	Hydrocephalus malresorptivus, post intraventricular hemorrhage	Hydrocephalus malresorptivus	No	Yes
**50**	135	Female	Yes	-0.08	Ventricular	CSF drainage	Hydrocephalus malresorptivus, post intraventricular hemorrhage	Hydrocephalus malresorptivus	No	Yes

**51**	136	Male	No	4.67	Ventricular	CSF drainage	Congenital aqueductal stenosis	Congenital aqueductal stenosis	No	Yes
**52**	137	Male	No	10.42	Ventricular	Arachnoid cyst drainage	Symptomatic arachnoid cyst	Symptomatic arachnoid cyst	No	No
**53**	139	Female	No	0.31	Ventricular	CSF drainage	Hydrocephalus malresorptivus, post-infectious	Hydrocephalus malresorptivus	No	Yes
**54**	139	Female	No	0.60	Ventricular	CSF drainage	Hydrocephalus malresorptivus, post-infectious	Hydrocephalus malresorptivus	No	Yes
**55**	140	Male	No	5.29	Ventricular	Tumor removal	Astrocytoma WHO III	Tumor	No	No
**56**	141	Female	Yes	-0.10	Ventricular	CSF drainage	Hydrocephalus malresorptivus, post intraventricular hemorrhage	Hydrocephalus malresorptivus	No	Yes
**57**	141	Female	Yes	-0.09	Ventricular	CSF drainage	Hydrocephalus malresorptivus, post intraventricular hemorrhage	Hydrocephalus malresorptivus	No	Yes
**58**	141	Female	Yes	-0.09	Ventricular	CSF drainage	Hydrocephalus malresorptivus, post intraventricular hemorrhage	Hydrocephalus malresorptivus	No	Yes
**59**	143	Male	No	9.89	Ventricular	CSF drainage	Teratoma III ventricle with hydrocephalus	Tumor	No	Yes
**60**	144	Male	No	3.33	Ventricular	CSF drainage	Hydrocephalus malresorptivus, post-infectious	Hydrocephalus malresorptivus	No	Yes
**61**	144	Male	No	4.82	Ventricular	CSF drainage	VP shunt dysfunction, meningitis	Hydrocephalus malresorptivus	Yes	Yes
**62**	147	Female	Yes	8.86	Spinal	Intrathecal baclofen pump	Tetraspastic cerebral palsy with prematurity, periventricular leukomalacia	Spastic cerebral palsy	No	No
**63**	147	Female	Yes	9.43	Spinal	Intrathecal baclofen pump	Tetraspastic cerebral palsy with prematurity, periventricular leukomalacia	Spastic cerebral palsy	No	No

Abbreviations: CSF, cerebrospinal fluid; VP ventriculoperitoneal shunt
