# Supplementary material for: Doublecortin and Glypican-2 concentrations in the cerebrospinal fluid from infants are developmentally downregulated
Source: PLoS One. 2023 Feb 17;18(2):e0279343. doi: 10.1371/journal.pone.0279343 (PMC9937498; doi:10.1371/journal.pone.0279343)
Supplement: S2 Table — (PDF) [file pone.0279343.s006.pdf]

S2 Table. Concentrations of DCX, GPC2, markers of neuronal damage and cytokines (in pg/ml) in each CSF sample from the 38 patients, along with corresponding age and main diagnosis.

Sample	Patient number	Adjusted age (years)	Main diagnosis	DCX	GPC2	NSE	S100b	IL-1β	IL-2	IL-4	IL-6	IL-8	IL-10	IL-13	IFN-γ	TNF-α
**1**	102	15.35	Ruptured arteriovenous malformation	<LOD	0.54	1336	NA	5.68	2.90	0.80	109	956	1.37	10.17	4.85	6.94
**2**	103	1.17	Posterior fossa ependymoma WHO II	<LOD	14.25	1806	1434	2.10	2.34	1.69	224	688	7.38	11.34	12.53	5.79
**3**	104	7.03	X-linked hydrocephalus syndrome	<LOD	26.03	7245	1210	<LOD	0.53	<LOD	0.93	60.25	0.35	<LOD	<LOD	0.39
**4**	106	17.91	Quadriplegic cerebral palsy with hypoxic encephalopathy	<LOD	15.10	2066	NA	0.06	0.14	<LOD	1.40	34.93	0.24	0.67	<LOD	<LOD
**5**	106	18.01	Quadriplegic cerebral palsy with hypoxic encephalopathy	<LOD	14.39	2676	1343	0.10	0.15	<LOD	3.03	39.67	0.29	0.75	<LOD	<LOD
**6**	107	-0.07	Congenital aqueductal stenosis	1115	111255	36118	1001	0.12	2.91	<LOD	1.70	49.54	0.08	0.78	<LOD	0.37
**7**	107	2.03	Congenital aqueductal stenosis	<LOD	4.82	537	2371	0.14	0.10	<LOD	12.18	32.74	<LOD	0.43	<LOD	<LOD
**8**	108	0.29	Symptomatic arachnoid cyst	<LOD	NA	5555	NA	NA	NA	NA	NA	NA	NA	NA	NA	NA
**9**	108	0.98	Symptomatic arachnoid cyst	<LOD	108	11084	341	0.35	0.94	0.04	5.59	74.52	0.32	2.09	2.04	2.24
**10**	109	10.04	Spastic cerebral palsy	<LOD	3.89	1716	NA	0.31	0.22	<LOD	1.18	26.32	<LOD	0.73	1.23	<LOD
**11**	109	10.35	Spastic cerebral palsy	<LOD	10.51	1936	704	0.20	0.23	<LOD	0.80	30.86	<LOD	0.63	<LOD	0.17

**12**	110	3.67	Hydrocephalus malresorptivus of unknown cause	<LOD	19.53	1396	721	0.06	0.08	<LOD	1.79	17.26	0.19	<LOD	<LOD	0.19
**13**	110	3.20	Hydrocephalus malresorptivus of unknown cause	<LOD	25.50	3286	2779	0.30	<LOD	<LOD	1.41	16.57	0.08	0.33	<LOD	<LOD
**14**	110	4.24	Hydrocephalus malresorptivus of unknown cause	<LOD	NA	167	NA	0.10	0.18	0.06	20.97	42.92	0.15	0.82	<LOD	<LOD
**15**	111	11.80	Symptomatic arachnoid cyst	<LOD	38.01	6105	1225	0.95	0.30	<LOD	1.92	74.28	0.25	1.71	1.03	0.39
**16**	113	2.87	Congenital aqueductal stenosis	<LOD	14.54	3836	NA	0.10	0.13	<LOD	3.39	17.59	0.24	0.46	1.32	<LOD
**17**	114	4.85	Langerhans Cell histiocytosis	<LOD	16.89	2676	NA	0.24	0.27	<LOD	13.36	57.93	0.31	1.66	<LOD	<LOD
**18**	116	7.20	Tectal glioma with hydrocephalus	<LOD	5.70	647	489	0.14	<LOD	<LOD	0.81	2.61	0.07	<LOD	0.20	<LOD
**19**	117	7.33	Tetraspastic cerebral palsy with perinatal asphyxic encephalopathy	<LOD	3.06	1286	456	0.05	0.12	<LOD	0.62	14.33	0.25	0.21	0.50	<LOD
**20**	117	7.40	Tetraspastic cerebral palsy with perinatal asphyxic encephalopathy	<LOD	3.62	1216	696	1.69	0.21	<LOD	1.01	28.61	0.20	<LOD	<LOD	<LOD
**21**	118	16.88	Spastic cerebral palsy with hypoxic encephalopathy	<LOD	34.75	4606	1595	0.31	0.32	<LOD	1.08	43.01	0.15	1.11	0.92	0.19
**22**	119	0.08	Hydrocephalus malresorptivus, post intraventricular hemorrhage	173	1500	5765	1889	15.61	2.96	2.76	878	644	7.67	12.51	20.47	<LOD
**23**	119	0.10	Hydrocephalus malresorptivus, post intraventricular hemorrhage	130	1800	5185	1450	2.44	0.94	0.14	30.92	157	1.88	3.02	2.82	1.82
**24**	119	0.20	Hydrocephalus malresorptivus, post intraventricular hemorrhage	10.06	602	3596	2312	1.67	0.89	2.13	309	263	1.07	5.64	2.13	2.32

**25**	120	1.86	Blake pouch cyst with hydrocephalus	<LOD	16.65	1426	1228	0.14	0.29	<LOD	2.62	54.64	0.24	0.98	1.36	0.46
**26**	121	0.02	Congenital aqueductal stenosis	704	23035	24900	966	0.16	1.40	<LOD	1.69	25.81	0.30	1.08	1.25	0.18
**27**	121	0.64	Congenital aqueductal stenosis	<LOD	38.53	1556	607	0.09	0.31	<LOD	3.58	14.32	0.20	0.55	0.89	<LOD
**28**	123	0.33	Symptomatic arachnoid cyst	<LOD	163	1676	1129	0.07	0.26	<LOD	1.06	11.51	0.10	0.31	<LOD	<LOD
**29**	124	1.82	Wound infection after spinal lipoma surgery	<LOD	17.60	3636	1437	96.73	7.22	25.13	6531	2721	62.02	36.35	32.08	37.96
**30**	125	-0.13	Hydrocephalus malresorptivus, post intraventricular hemorrhage	1244	59621	24860	1588	1.56	2.91	0.12	8.56	701	0.89	8.53	5.39	4.94
**31**	125	0.09	Hydrocephalus malresorptivus, post intraventricular hemorrhage	190	3516	13713	1444	13.47	4.48	1.93	249	1145	8.05	12.14	16.48	4.81
**32**	126	0.05	Myelomeningocele with hydrocephalus	212	3879	9087	532	0.07	0.91	0.0005	2.49	27.76	0.22	<LOD	<LOD	<LOD
**33**	127	2.62	Bilateral hygroma post epilepsy surgical hemispherotomy	<LOD	7.04	645	404	0.07	0.37	0.26	44.93	58.69	0.33	1.56	<LOD	0.73
**34**	128	8.32	Suspected hereditary spastic paraparesis	<LOD	6.13	2136	482	0.08	0.14	0.08	1.06	22.03	0.13	0.79	<LOD	<LOD
**35**	128	8.32	Suspected hereditary spastic paraparesis	<LOD	5.99	2700	278	0.03	0.03	<LOD	0.92	19.99	<LOD	<LOD	<LOD	<LOD
**36**	129	0.23	Crouzon syndrome	<LOD	19.88	1636	1826	<LOD	<LOD	<LOD	0.68	3.14	<LOD	<LOD	<LOD	<LOD
**37**	129	0.38	Crouzon syndrome	<LOD	13.77	858	847	0.02	<LOD	<LOD	0.72	5.28	0.11	<LOD	<LOD	<LOD
**38**	130	3.52	Tectal glioma with hydrocephalus	<LOD	36.42	10578	13480	0.09	0.07	0.05	2.90	36.14	0.05	<LOD	<LOD	<LOD

**39**	131	0.61	Crouzon syndrome	<LOD	44.78	1719	831	0.04	<LOD	0.10	26.43	21.76	<LOD	0.56	<LOD	0.25
**40**	132	0.53	Congenital communicating hydrocephalus	<LOD	164	3765	2818	0.2	0.53	0.10	2.53	104	0.34	2.04	<LOD	0.80
**41**	133	7.85	Spastic cerebral palsy with perinatal hypoxic encephalopathy	<LOD	NA	2941	7.00	NA	NA	NA	NA	NA	NA	NA	NA	NA
**42**	133	8.73	Spastic cerebral palsy with perinatal hypoxic encephalopathy	<LOD	12.67	3700	445	0.2	0.39	0.04	2.71	83.29	0.28	1.82	2.29	0.66
**43**	134	5.83	Spastic cerebral palsy with perinatal intraventricular hemorrhage and hydrocephalus	<LOD	101	9226	1671	0.2	0.66	0.08	0.77	76.77	0.31	1.75	1.99	0.73
**44**	135	-0.28	Hydrocephalus malresorptivus, post intraventricular hemorrhage	2992	NA	34524	NA	NA	NA	NA	NA	NA	NA	NA	NA	NA
**45**	135	-0.26	Hydrocephalus malresorptivus, post intraventricular hemorrhage	2957	58881	35302	1480	7.7	4.12	0.56	19.41	2254	2.39	11.15	3.18	12.22
**46**	135	-0.25	Hydrocephalus malresorptivus, post intraventricular hemorrhage	2480	43984	24592	1317	6.9	3.81	1.26	13.64	2136	1.37	13.33	3.03	6.15
**47**	135	-0.24	Hydrocephalus malresorptivus, post intraventricular hemorrhage	1745	39095	21556	1757	4.8	3.33	0.89	8.72	1614	1.38	9.13	4.01	4.77
**48**	135	-0.24	Hydrocephalus malresorptivus, post intraventricular hemorrhage	1602	43408	21769	1829	4.1	3.57	0.34	6.78	1384	1.06	7.51	2.62	4.63
**49**	135	-0.10	Hydrocephalus malresorptivus, post intraventricular hemorrhage	646	34370	21454	838	0.7	2.04	0.15	3.29	172	0.27	3.23	1.67	0.87
**50**	135	-0.08	Hydrocephalus malresorptivus, post intraventricular hemorrhage	735	22030	20380	1529	0.3	1.58	0.07	2.23	86.75	0.19	1.83	0.79	0.57

**51**	136	4.67	Congenital aqueductal stenosis	<LOD	3.93	1099	1706	<LOD	<LOD	<LOD	3.21	3.45	<LOD	<LOD	<LOD	<LOD
**52**	137	10.42	Symptomatic arachnoid cyst	<LOD	4.32	2006	1879	0.03	<LOD	<LOD	3.62	15.29	0.11	<LOD	<LOD	<LOD
**53**	139	0.31	Hydrocephalus malresorptivus, post-infectious	63.82	1374	4820	2849	0.6	2.27	0.40	4.24	443	4.25	4.51	12.13	2.92
**54**	139	0.60	Hydrocephalus malresorptivus, post-infectious	20.53	799	22991	9514	0.5	1.92	0.28	36.99	41.29	0.81	1.65	39.54	2.21
**55**	140	5.29	Astrocytoma WHO III	254	36.19	46512	297795	0.5	0.50	0.17	6.80	76.20	0.49	2.28	2.29	6.52
**56**	141	-0.10	Hydrocephalus malresorptivus, post intraventricular hemorrhage	4861	45430	32590	18598	10.8	3.18	1.11	88.82	3211	3.64	13.45	7.56	12.47
**57**	141	-0.09	Hydrocephalus malresorptivus, post intraventricular hemorrhage	9119	52828	59758	15387	20.4	4.84	2.23	93.93	5049	3.19	15.71	10.11	11.91
**58**	141	-0.09	Hydrocephalus malresorptivus, post intraventricular hemorrhage	9130	49397	66941	14472	41.1	8.21	7.32	652	11051	9.86	24.60	26.61	248
**59**	143	9.89	Teratoma III ventricle with hydrocephalus	329	19.67	2321	1370	0.2	0.37	0.13	4.48	77.52	0.50	2.10	2.31	0.84
**60**	144	3.33	Hydrocephalus malresorptivus, post-infectious	<LOD	26.69	27110	30634	0.2	0.51	0.12	0.99	54.64	0.45	1.65	1.94	0.43
**61**	144	4.82	VP shunt dysfunction, meningitis	4.13	32.97	136179	NA	NA	NA	NA	NA	NA	NA	NA	NA	NA
**62**	147	8.86	Tetraspastic cerebral palsy with prematurity, periventricular leukomalacia	<LOD	11.51	2163	612	0.3	0.72	0.31	2.85	58.70	0.61	<LOD	5.89	<LOD
**63**	147	9.43	Tetraspastic cerebral palsy with prematurity, periventricular leukomalacia	<LOD	9.95	1502	950	0.3	0.75	0.22	1.86	48.36	0.67	<LOD	5.09	<LOD

Abbreviations: CSF, cerebrospinal fluid; VP ventriculoperitoneal shunt; NA, sample not available (i.e., used up in previous assays); <LOD: value below limit of detection within assay plate
