# Supplementary material for: Doublecortin and Glypican-2 concentrations in the cerebrospinal fluid from infants are developmentally downregulated
Source: PLoS One. 2023 Feb 17;18(2):e0279343. doi: 10.1371/journal.pone.0279343 (PMC9937498; doi:10.1371/journal.pone.0279343)
Supplement: S3 Table — (PDF) [file pone.0279343.s007.pdf]

S3 Table. Data availability of variables and summary statistics including all measurements from each patient.

Analyte	Total no. of CSF samples available	No. of measurements above detection limit	Median concentration (pg/ml)	Interquartile range (pg/ml)
**DCX**	63	23	704.38	181.57 to 2112.47
**GPC2**	59	59	33	13.5 to 1650
**NSE**	63	63	3700	1717.5 to 20917
**S100B**	54	54	1402	748.5 to 1866.5
**IL-1β**	59	56	0.26	0.1 to 1.68
**IL-2**	59	52	0.59	0.25 to 2.48
**IL-4**	59	34	0.24	0.1 to 1.06
**IL-6**	59	59	3.21	1.4 to 16.52
**IL-8**	59	59	54.64	26.07 to 217.64
**IL-10**	59	52	0.31	0.2 to 1.15
**IL-13**	59	46	1.78	0.78 to 8.28
**IFN-γ**	59	36	2.47	1.35 to 6.3
**TNF-α**	59	35	1.82	0.44 to 5.36
