# Supplementary material for: Doublecortin and Glypican-2 concentrations in the cerebrospinal fluid from infants are developmentally downregulated
Source: PLoS One. 2023 Feb 17;18(2):e0279343. doi: 10.1371/journal.pone.0279343 (PMC9937498; doi:10.1371/journal.pone.0279343)
Supplement: S4 Table — (PDF) [file pone.0279343.s008.pdf]

S4 Tables.

Parameters of the asymptotic regression model assessing the relationship between Doublecortin and adjusted age.

	Doublecortin			
	**Estimate**	**l-95% CrI**	**u-95% CrI**	$\hat{R}$
Asym	-7.9	-20.4	-1.8	1.0
R0	6.3	5.6	7.0	1.0
lrc	0.3	-0.5	1	1.0

$\hat{R}$: Gelman-Rubin convergence diagnostic

Parameters of the asymptotic regression model assessing the relationship between Glypican-2 and adjusted age.

	Glypican-2			
	**Estimate**	**l-95% CrI**	**u-95% CrI**	$\hat{R}$
Asym	2.6	2.0	3.1	1.0
R0	8.6	7.2	9.9	1.0
lrc	-3.4	-3.7	-3.0	1.0

$\hat{R}$: Gelman-Rubin convergence diagnostic

Parameters of the linear regression model assessing the relationship between Doublecortin and Glypican-2.

	Doublecortin			
	**Estimate**	**l-95% CrI**	**u-95% CrI**	$\hat{R}$
**Glypican-2**				
Intercept	-5.05	-8.21	-2.77	1.0
Log Glypican-2	1.15	0.86	1.51	1.0
