# Supplementary material for: Doublecortin and Glypican-2 concentrations in the cerebrospinal fluid from infants are developmentally downregulated
Source: PLoS One. 2023 Feb 17;18(2):e0279343. doi: 10.1371/journal.pone.0279343 (PMC9937498; doi:10.1371/journal.pone.0279343)
Supplement: S5 Table — (PDF) [file pone.0279343.s009.pdf]

S5 Tables.

Parameters of the univariable linear regression models assessing the relation between Doublecortin and each clinical variable.

	Estimate	l-95% CrI	u-95% CrI	$\widehat{R}$	Significant
**Sex**					
Intercept (Reference: Female)	-0.36	-3.97	2.37	1.00	
Male	-1.35	-5.80	3.03	1.00	
**Infections [Yes / No]**					
Intercept (Reference: No)	-1.10	-4.23	1.10	1.00	
Yes	1.20	-2.31	4.77	1.00	
**Hydrocephalus [Yes / No]**					
Intercept (Reference: No)	-5.23	-11.43	-1.00	1.00	
Yes	6.15	1.50	12.23	1.00	*
**IL-1β**					
Intercept	-0.43	-4.23	2.10	1.00	
Log IL-1β	0.83	0.08	1.67	1.00	*
**IL-2**					
Intercept	0.69	-2.18	2.63	1.00	
Log IL-2	2.45	1.56	3.63	1.00	*
**IL-4**					
Intercept	-0.06	-4.47	2.92	1.00	
Log IL-4	-0.02	-0.50	0.47	1.00	
**IL-6**					
Intercept	-2.42	-7.02	0.71	1.00	
Log IL-6	0.07	-0.70	0.93	1.00	
**IL-8**					
Intercept	-6.13	-11.36	-2.00	1.00	
Log IL-8	1.17	0.36	2.11	1.00	*
**IL-10**					
Intercept	-0.75	-4.96	1.96	1.00	
Log IL-10	0.68	-0.08	1.50	1.00	
**IL-13**					
Intercept	-1.77	-5.55	0.82	1.00	
Log IL-13	1.73	0.54	3.08	1.00	*
**IFN-γ**					
Intercept	-0.93	-5.13	1.94	1.00	
Log IFN-γ	0.81	-0.22	1.96	1.00	
**TNF-α**					
Intercept	0.10	-3.74	2.62	1.00	
Log TNF-α	0.42	-0.07	0.99	1.00	
**NSE**					
Intercept	-19.51	-29.86	-12.04	1.00	
Log NSE	2.24	1.44	3.31	1.00	*
**S100B**					
Intercept	-8.48	-20.58	1.77	1.00	
Log S100B	0.95	-0.44	2.39	1.00	

$\widehat{R}$: Gelman-Rubin convergence diagnostic
l-95% CI: lower 95 credible interval
u-95% CI: upper 95 credible interval

Parameters of the univariable linear regression models assessing the relation between Glypican 2 and each clinical variable.

	Estimate	l-95% CrI	u-95% CrI	$\widehat{R}$	Significant
**Sex**					
Intercept (Reference: Female)	4.7	3.4	6.0	1.00	
Male	-1.1	-3.0	0.8	1.00	
**Infections [Yes / No]**					
Intercept (Reference: No)	4.2	3.2	5.2	1.00	
Yes	-0.4	-2.8	2.1	1.00	
**Hydrocephalus [Yes / No]**					
Intercept (Reference: No)	2.7	1.2	4.2	1.00	
Yes	2.4	0.5	4.2	1.00	*
**IL-1β**					
Intercept	4.9	3.7	5.9	1.00	
Log IL-1β	0.5	0.02	1.0	1.00	*
**IL-2**					
Intercept	5.4	4.4	6.3	1.00	
Log IL-2	1.6	1.1	2.2	1.00	*
**IL-4**					
Intercept	4.5	2.9	6.0	1.00	
Log IL-4	-0.2	-0.5	0.1	1.00	
**IL-6**					
Intercept	4.1	2.9	5.3	1.00	
Log IL-6	0.1	-0.4	0.5	1.00	
**IL-8**					
Intercept	1.0	-1.1	3.0	1.00	
Log IL-8	0.8	0.3	1.2	1.00	*
**IL-10**					
Intercept	4.6	3.4	5.7	1.00	
Log IL-10	0.3	-0.3	0.8	1.00	
**IL-13**					
Intercept	4.0	2.8	5.1	1.00	
Log IL-13	1.0	0.2	1.8	1.00	*
**IFN-γ**					
Intercept	3.9	2.3	5.5	1.00	
Log IFN-γ	0.5	-0.3	1.4	1.00	
**TNF-α**					
Intercept	4.9	3.6	6.3	1.00	
Log TNF-α	0.2	-0.2	0.6	1.00	
**NSE**					
Intercept	-10.4	-13.5	-7.3	1.00	
Log NSE	1.7	1.4	2.1	1.00	*
**S100B**					
Intercept	2.1	-3.4	7.5	1.00	
Log S100B	0.3	-0.4	1.0	1.00	

$\widehat{R}$: Gelman-Rubin convergence diagnostic
l-95% CI: lower 95 credible interval
u-95% CI: upper 95 credible interval
