# Supplementary material for: Doublecortin and Glypican-2 concentrations in the cerebrospinal fluid from infants are developmentally downregulated
Source: PLoS One. 2023 Feb 17;18(2):e0279343. doi: 10.1371/journal.pone.0279343 (PMC9937498; doi:10.1371/journal.pone.0279343)
Supplement: S1 File — (PDF) [file pone.0279343.s010.pdf]

S1 Supporting Information on statistical methods.

Asymptotic regression model formulation

Asymptotic regression function can be used to model the decrease of concentration over time, where concentration levels approach a horizontal asymptote (plateau). In our study, the asymptotic function is defined as:

$$Y = Asym + (R0 - Asym) * e^{-e^{-lrc} * t}$$

where *Asym* describes the minimal attainable value for *Y*, *R0* is the initial value at time 0 (term birth), *lrc* is the natural logarithm of the rate of decrease and *t* is time.

Based on this asymptotic function we have built our model as follows. We defined Y_{it} as the sample concentration of DCX and GPC2 for each patient $i \ (i \ in \ 1, \ldots, N)$ at time point $t \ (t \ in \ 1, \ldots, T)$. We assumed Y_{it} to follow a normal distribution $Y_{it} = N(\mu_{it}, \sigma^2)$, where $\mu_{it} = Asym_i + (R0_i - Asym_i) * e^{-e^{-lrc_i} * t} + \varphi_i$.
Asym, R0, and *lrc* describe the parameters of the asymptotic function and φ_i is a random effect that takes into account the variation on the individual level.

We considered the following values for the parameters of the uninformative prior distribution. *Asym* = $N(2.5; 100)$, *R0* = $N(12; 100)$ and *lrc* = $N(-3; 100)$. We used a self-starting asymptotic regression model to determine the hyperparameter μ of the prior distributions.
φ_i is a random effect that is assumed to have a prior of $Student - t(3; 0; \ 2.5)$.

In all models including measurements of DCX, we had measurements below the detection limit of the assay. Using the fully probabilistic Bayesian approach, we could integrate these censored values out instead of imputing them.
The probability of each censored data point is

$$\Pr[y > U] = \int_U^{\infty} normal\ (y \mid \mu, \sigma) dy = 1 - \phi(\tfrac{y-\mu}{\sigma}), \qquad [1]$$

and the total probability of M censored observations on the log scale is

$$log \prod_{m=1}^{M} \Pr[y_m > U] = \log (1 - \phi\left(\tfrac{y-\mu}{\sigma}\right))^M, \qquad [1]$$

where $\phi()$ is the standard normal cumulative distribution function and M the number of censored observations.

Linear regression model formulations

To assess a potential linear relationship between DCX and GPC2 with conventional markers of brain damage and (NSE, S100B) and inflammation (IL-1β, IL-2, IL-4, IL-6, IL-8, IL-10, IL-13, IFN-γ, TNF-α) we fitted a univariable linear mixed-effects model with a random intercept to account for the variation on the individual level.

In our model, we defined Y_{it} as the sample concentration of DCX and GPC2 for each patient i $(i\ in\ 1, \ldots, N)$ at time point t $(t\ in\ 1, \ldots, T)$. We assumed Y_{it} to follow a normal distribution $Y_{it} = N(\mu_{it}, \sigma^2)$, where $\mu_{it} = \alpha + \beta(Time_{it}) + \varphi_i$.
We considered the following values for the parameters of the prior distribution. For α (intercept) and β (slope), we used $N(0; 10)$ as an uninformative prior distribution. φ_i is a random effect that is assumed to have a prior of $Student - t(3; 0;\ 2.5)$.

Implementation details

To estimate the posterior probability of the parameters, STAN uses a Markov chain Monte Carlo (MCMC) algorithm with a No-U-Turn Sampler (NUTS). We sampled four chains with 10,000 iterations each; in each chain, we use 5,000 iterations as a warm-up period.

Reference:

[1] Team SD. Stan User's Guide, version 2.23.2020.
