# Supplementary material for: Doublecortin and Glypican-2 concentrations in the cerebrospinal fluid from infants are developmentally downregulated
Source: PLoS One. 2023 Feb 17;18(2):e0279343. doi: 10.1371/journal.pone.0279343 (PMC9937498; doi:10.1371/journal.pone.0279343)

S2 Supporting Information on the sensitivity analyses for the relations between CSF-Doublecortin/Glypican-2 and NSE.

Sensitivity analysis – Exclusion of blood contaminated samples

Since hemolysis is a well-documented confounder of NSE determination, we ran a sensitivity analysis, where we excluded all samples with visible hemolysis from our analysis to assess the relationship between Doublecortin and Glypican-2 with NSE.

Relationship between Doublecortin and NSE

Parameters of the linear regression model assessing the relationship between doublecortin and NSE, after exclusion of blood contaminated samples (N = 47).

	Doublecortin			
	**Estimate**	**l-95% CI**	**u-95% CI**	$\hat{R}$
**NSE**				
Intercept	-27.8	-49.58	-13.4	1.0
Log NSE	3.11	1.51	5.45	1.0

$\hat{R}$: Gelman-Rubin convergence diagnostic
l-95% CI: lower 95 credible interval
u-95% CI: upper 95 credible interval

Graph showing Log concentration of doublecortin (DCX) by Log concentration of NSE with linear mixed-effect regression model (blue line) and 95% credible interval (grey area). The blue and red dots are the log transformed CSF-DCX values. Blue dots represent values over, and red dots below the limit of detection.

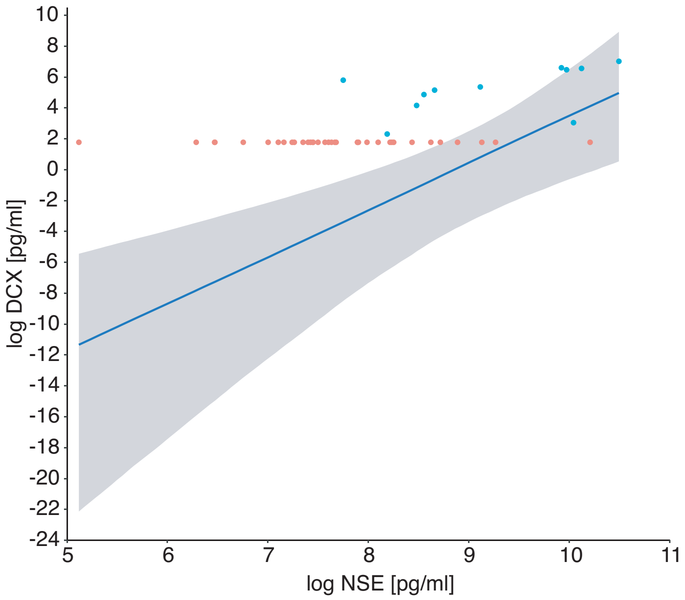

Relationship between Glypican-2 and NSE

Parameters of the linear regression model assessing the relationship between Glypican-2 and NSE, after exclusion of blood contaminated samples (N = 44).

	Glypican-2			
	**Estimate**	**l-95% CI**	**u-95% CI**	$\hat{R}$
**NSE**				
Intercept	-11.47	-14.93	-7.91	1.0
Log NSE	1.89	1.46	2.32	1.0

$\hat{R}$: Gelman-Rubin convergence diagnostic
l-95% CI: lower 95 credible interval
u-95% CI: upper 95 credible interval

Graph showing Log concentration of Glypican-2 (GPC2) by Log concentration of NSE with a linear mixed-effect regression model (blue line) and 95% credible interval (grey area).

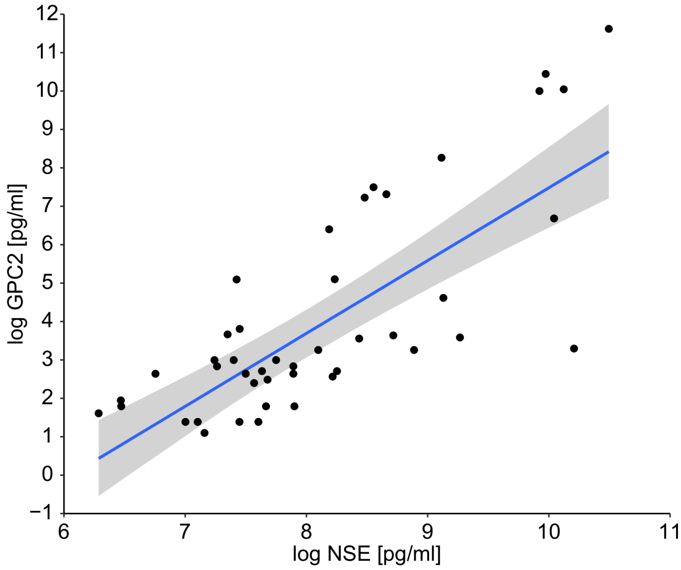

Supplement: S2 File — (PDF) [file pone.0279343.s011.pdf]
