# Supplementary material for: Doublecortin and Glypican-2 concentrations in the cerebrospinal fluid from infants are developmentally downregulated
Source: PLoS One. 2023 Feb 17;18(2):e0279343. doi: 10.1371/journal.pone.0279343 (PMC9937498; doi:10.1371/journal.pone.0279343)
Supplement: Attachment — Submitted filename: Response to Reviewers.docx [file pone.0279343.s012.docx]

Reviewer #1: Neurogenesis plays a key role on brain development and homeostasis but there are not currently good indicators of the neurogenic state in a living animal. The cerebrospinal fluid, CSF, may reflect the physiological state of the central nervous system, as shown by the latest findings reporting cognitive benefits to adult mice after infusion of CSF from young mice.

Doublecortin (DCX) and Glypican-2 (GPC2) are key neurodevelopmental proteins involved in neurogenesis in mice, and have been proposed as potential reporters of neurogenesis. DCX and GPC2 have peak expression in the CSF perinatally and decline subsequently in early postnatal stages in rodents, but there are few or no reports on the CSF expression of those markers in humans.

Data for human patients is key to understanding human development and disease, but ethical experimental constraints limit their availability. This highlights the relevance of the present study by Guzman and colleagues, an approach to systematically investigate DCX and GPC2 expression in infant CSF. The same constraints affect this study, as data provided belongs to pediatric patients and is, therefore, partial and reflects infants requiring neurosurgery. But the information provided, together with future similar studies will help understand human neurogenesis.

The biggest strength of the study is the observation that human CSF expression of both factors reflects experimental data from rodents. The manuscript is well written and worthy of publication in PLOS ONE, with the authors nicely acknowledging the weak points of the study in the discussion. The reviewer would nevertheless suggest a few minor modifications to provide all available data to the public. In the reviewer’s view, this would improve the impact of the manuscript.

We are very thankful to the reviewer for providing a thorough report on our study, and for its overall positive assessment. We are also grateful for the insightful comments. Please find below our point-by-point response.

Major Points.

1. Full details of the clinical baseline characteristics for each patient are accounted for in the Supplementary Table 1, yet the information regarding the individual patient’s values for the DCX and GPC2, as well as the values for cytokines and markers for brain damage are not provided. In view of the reviewer, the information regarding the patient diagnosis accompanied by the individual measurements of DCX, GPC2 and the relationship with the individual values of the rest of molecules tested could provide a better understanding of their role.

We agree with the reviewer that these critical data were missing. Accordingly, we have now provided a new supporting Table 2 (S2 Table) in which concentrations of all analytes in each CSF sample are reported, along with age and main diagnosis of the patients. The relationship between CSF-DCX, CSF-GPC2 and each of these analytes is reported in the result section pp. 13-14, summarized in Table 3 and Table S5, and discussed pp.16-18.

2. Similarly, some of the patients were subject to repeated measures, but the respective measurements are not accounted for in the presented manuscript. In the reviewer’s opinion, this longitudinal information should be provided, as it would allow for better characterization of the dynamics of doublecortin and glypican-2 during human development, and in disease.

It is correct that in the main manuscript, we present the median concentration and interquartile range calculated using values at first measurement. This is meant to avoid misrepresenting the population by giving too much weight to values from patients with repeated sampling. The summary statistics using all available values are nevertheless provided in Supporting Table 3 (S3 Table).

Please note that for the assessment of the relationships between CSF-DCX, CSF-GPC2 and all other parameters, all values from each patient were taken into account, and not just values at first measurements. These two points are now made clear in the Method section pp. 7-8, lines 161-166.

We agree with the reviewer that the longitudinal CSF concentrations of DCX and GPC2 in patients who underwent repeated samplings are missing. Such graphs are now provided in Fig 3 and S1 Fig. Overall, as predicted by our model, CSF-DCX and CSF-GPC2 decline over time in the same patients. Patients for whom first sampling occurred before predicted time of birth, and second sampling months or years after (e.g., patients 125 and 107) illustrate well this asymptotic age-related decline. The stabilization of CSF-GPC2 from one year onward is also illustrated in patients 110, 144, 117, 147, 109 and 106, whose CSF-GPC2 shows only small variations. An increase in CSF-DCX and GPC2 is nevertheless observed in patient 141.This may be the consequence of CSF removal by serial tapping of ventricular reservoir within a very short time-lapse (three days), as described in the literature in the context of repeated CSF lumbar puncture (PMID: 31831030), which may disturb CSF dynamics. Please note that we did not include graphs for patients 108, 128 and 133: patients 108 and 133 had CSF-DCX values below limit of detection (LOD) and only one available CSF-GPC2 value, due to unavailability of the second CSF sample. Patient 128 had two CSF samplings the same day. It was therefore not possible to display an age-related graph for these 3 patients. These data are now presented the Result section, pp. 12-13, and discussed in a new paragraph p. 15.

3. The reviewer wonders whether attributing the DCX values below the detection limits to the mean values of the lower detection limit to DCX concentration is generating an artifact to the data. Indeed, to the reviewer’s eye, the DCX data matches better a linear regression than an asymptote once the values that are below the detection limits of the assay (or data from patients with diseases of proliferative nature) are left out of the analysis. Would a randomized attribution of the values of DCX concentration –from 0 pg/ml to the lower detection limit– alter the interpretation of the data? Alternatively, a visual indication of the actual measurement should be included in the graph.

Thank you for this comment. We agree with the reviewer, imputing the censored DCX values below LOD with the mean LOD, or any other fixed value, would introduce bias to the data. Using the Bayesian approach in our analysis, we do not need to impute or discard any of the censored values. Instead, the censored values can be included into the model, where they are sampled together with the other parameters in the model, such that the censored data will finally get integrated out of the posterior distribution.

We have added a detailed description in S1 Supporting Information and have clarified it in the manuscript (p. 8): “*In all models including measurements of DCX, we had measurements below the LOD of the assay. Using the fully probabilistic Bayesian approach, it is not necessary to ignore or to impute these censored values; instead, they can be incorporated into the model, where they are eventually integrated out of the posterior distribution [23]. For better visualization, in the graphs where DCX data are reported, the censored values are displayed using the mean value of the LOD of all DCX assays, i.e., 5.8 pg/ml.*”

4. The researchers found positive correlation between the CSF levels of DCX and GPC-2 and the cytokines IL-1beta, IL-2, IL-8 and IL-13, and the marker for brain damage NSE. The authors provide a graph showing that log-transformed measurements of NSE do not show an asymptotic decline with age. The authors state that a visual assessment of the relation between log-transformed concentrations of cytokines with age indicated an absence of relation between those parameters. The authors provide the graphs for IL-1β, IL-2, IL-8 and IL-13. To the reviewer’s eye, these show that maximum expression levels appear perinatally to later stabilize. On the other hand, the authors do not display the same data for the remaining interleukins and the marker S100B. In view of the reviewer, these graphs should also be provided.

Thank you for this very insightful comment. We have now withdrawn our statement that “*The relation between log-transformed concentrations of cytokines and age was also examined, but visual assessment of graphs indicated absence of relation between these parameters*”. As the reviewer correctly points out, visual assessment does seem to indicate that CSF concentrations of IL-1β, IL-2, IL-8 and IL-13 decline with age with higher concentrations during the perinatal stages followed by a decrease and stabilization at later ages. Therefore, to substantiate this “visual impression”, we have now tested whether any of these 4 cytokines fits into the pre-specified asymptotic model that we applied for DCX, GPC2 and NSE.

Our results indicate that the CSF concentration of IL-2 over time fits into the asymptotic model (new S3 Fig), while the CSF levels of IL-1β, IL-8 and IL-13 do not. This new piece of data is now included into the manuscript p. 14 and discussed p. 18. A relation between CSF concentrations of IL-1β, IL-8 and Il-13 and age still remains plausible though, but we did not investigate further the nature of that relationship, as it is beyond the scope of the present study.

We also agree with the reviewer that graphs showing the concentrations of IL-4, IL-6, IL-10, IFN-γ, TNF-α and S100B as a function of age were lacking. These graphs are now provided in S4 Fig, together with the graphs for IL-1β, IL-8 and IL-13. Concerning IL-4, IL-6, IL-10, IFN-γ, TNF-α and S100B, it is actually clear that the concentration of these cytokines in the CSF do not follow a particular age-related pattern.

Minor Points.

1. A below-detection limit value is present in the figure-1 graph.

We thank the reviewer for pointing this out, as this may be confusing to the reader. That particular point corresponds to a value that was below the mean LOD of all assays, but still above the detection limit in the particular assay. We have now added a sentence in the legend of figure 1 to clarify this point p. 12.

2. S2 table is labelled as “S1”. S4 tables are labelled as “S3”.

Indeed, this was a mistake and it has now been corrected.
